# SARS-CoV-2 Seroprevalence at an Urban Hospital in Haiti

**DOI:** 10.7759/cureus.27690

**Published:** 2022-08-04

**Authors:** Robert Price, Jeffrey Cho, Scott Nelson

**Affiliations:** 1 Medicine, Loma Linda University School of Medicine, Loma Linda, USA; 2 Primary Care, Hôpital Adventiste d'Haïti, Port-au-Prince, HTI; 3 Family Medicine, Loma Linda University Health, Loma Linda, USA; 4 Orthopedics, Hôpital Adventiste d'Haïti, Port-au-Prince, HTI; 5 Orthopedics, Loma Linda University Health, Loma Linda, USA

**Keywords:** low-to-middle-income country, haiti, seroprevalence, covid-19, sars-cov-2

## Abstract

Background: Much to the surprise of the global community, Haiti has had far fewer COVID-19 cases and deaths than initially expected. In this study, we sought to estimate the seroprevalence of COVID-19 in a convenience cohort based in Port-au-Prince, Haiti, to elucidate potential reasons for the apparently low burden of COVID-19 in Haiti.

Methods: We performed a cross-sectional analysis of SARS-CoV-2 antibody prevalence in patients aged one to 89 years old who were seen at the Haiti Adventist Hospital (HAH) laboratory between December 17, 2020, and July 3, 2021, with an order requiring a blood draw. We excluded patients outside of the age range and those who did not verbally consent to the study. We tested residual patient serum samples using the Biosys PlusTM COVID-19 IgM/IgG Rapid Test.

Findings: Of 9,740 patients seen by the HAH laboratory from December 2020 to July 2021, 538 consented to have antibody testing and answer survey questions. 529 were included in the final analysis. We excluded nine participants who were aged greater than 89 (n=3), aged less than one (n=2), or had results that were not properly recorded (n=4). Three of the tested patients were repeat testers, with one who had been tested three times. These repeat results were included for a final seroprevalence analysis of 533 samples. In the final participant pool, 142 (26.6%) of 533 samples tested positive for either IgM, IgG, or both antibodies to the SARS-CoV-2 virus. Adjustment for test sensitivity resulted in an estimated seroprevalence of 28.7% (95% CI 24.9-32.9). We observed significant differences in seroprevalences among age groups, with seroprevalence increasing with age.

Interpretation: The SARS-CoV-2 antibody seroprevalence in Haiti appears to be greater than the publicly reported statistics by several orders of magnitude. Furthermore, a reduced fatality rate relative to high-income countries points to uncertain factors that may confer immunologic resistance in the Haitian population.

## Introduction

The first two cases of Severe Acute Respiratory Syndrome Coronavirus 2 (SARS-CoV-2) in Haiti were confirmed on March 19, 2020 [1]. Given a porous border with the highly affected Dominican Republic [2], limited access to hospital beds, lack of clean water and sanitation, densely populated slums controlled by gangs, and a pervasive poverty that affects nearly 70% of households, the virus was expected to have a devastating impact [3]. In response to the confirmation of COVID-19 in the country, the Haitian government immediately declared a state of emergency that provided for the temporary closure of schools and universities, the closure of manufacturing plants, curfew establishments, a ban on public gatherings of more than ten people, in addition to shutting down all modes of human transport entering the country [1]. Over the course of the pandemic, adherence to social distancing and other infection prevention measures has been low. Sheltering in place and commercial lockdowns are not an option for a vast majority of the population, many of whom make less than $2 a day [4]. Stigmatization against COVID-19 and vaccine hesitancy remains high, with less than 1% of the population fully vaccinated since vaccines first arrived in the country on July 14, 2021 [5].

Despite Haiti’s persistent vulnerabilities and compounding natural and man-made disasters, the grim predictions regarding COVID-19 have not been nearly as devastating as anticipated. As of January 1, 2022, Haiti has reported 25,985 total cases and 766 deaths since March 19, 2020 [6]. The Dominican Republic, which shares the island of Hispaniola with Haiti and has a similar population size, comparison, reported 419,927 total cases and 4,247 deaths since March 1, 2020 [7]. The apparent transmissibility of SARS-CoV-2 and the number of COVID-related deaths per 100,000 people have been much lower in Haiti compared to the Dominican Republic and other countries with better healthcare resources [5]. While the apparent attenuation of SARS-CoV-2 in Haiti may be partly due to inadequate testing [8], it is unlikely that this is the sole explanation given the dramatic difference in the death rate.

One strategy used to explain unique COVID-19 responses in various parts of the world is to use seroprevalence surveys to complement standard RT-PCR testing used to confirm symptomatic cases [9]. This method is beneficial since it can provide an estimate of prevalence that includes asymptomatic and minimally symptomatic individuals. In addition, this method provides a convenient means, if done serially, to assess the transmission of the pandemic over time and the proportion of the population that has attained natural immunity from the virus. After infection with SARS-CoV-2, it is estimated that immunologic memory through SARS-CoV-2 specific antibodies, CD4+ T cells, CD8+ T cells, and memory B-cells lasts from five to eight months depending on the cell lineage [10]. This immunity is likely to provide substantial protection from reinfection despite recent evidence that antibody responses during COVID-19 vaccination provide superior neutralization of circulating variants [11,12]. Seroprevalence studies throughout regions most affected by COVID-19 have provided seropositivity ranges that vary according to the aggressiveness of the region’s COVID-19 response, human development indices, relative average income, and geographic latitude and/or climate. For example, in early-to-mid-2020, high-income regions such as Los Angeles County, the United States; Switzerland; and Spain had estimated seroprevalence values of 4.65%, 7.85%, and 5%, respectively [13-15]. In contrast, during a similar time period, low-income countries were found to have varying levels of seroprevalence among specific populations, including asymptomatic health care workers in Nigeria (45.1%) and Malawi (12.3%), blood donors in Kenya (4.3%), and a population-based household survey in Zambia (10.6%) [16-19]. Reported seroprevalence rates in Port-au-Prince from mid-2020 to early 2021 ranged from 9.1% to 11% found in a preprint article to 39% in a published article [20-21]. ­While these early estimates are a useful starting point, further reports will be necessary to establish trends.

In this study, we aimed to estimate SARS-CoV-2 seroprevalence among the patients who were having labs drawn at Haiti Adventist Hospital (HAH). HAH is a 55-bed, faith-based hospital located in Carrefour, a commune of Port-au-Prince and home to more than a half million people. This study was done at HAH with support from Loma Linda University. We aimed to estimate the seroprevalence of SARS-CoV-2 to inform policy and provide guidance for proper precautions and prioritization of healthcare resources.

## Materials and methods

Study design and participants

We performed a cross-sectional analysis of patients aged one to 89 years old who were seen at the HAH laboratory between December 17, 2020 and July 3, 2021 with an order requiring a blood draw. We excluded people aged less than one year old or greater than 89 years old. HAH is part of the Adventist health care system and has a longstanding partnership with Loma Linda University. The study was approved by the institutional review board of Loma Linda University Health. Designated laboratory staff asked eligible patients if they would be willing to participate in the research study and answer survey questions. Verbal consent was obtained from all adult patients and from the parents or representatives of all patients aged younger than 20 years. Participants’ data were coded to protect their identity. Study forms and data were protected and handled exclusively by the study researchers.

Procedures

After consent was obtained using a verbal consent script, the laboratory worker gathered information on demographics, comorbidities, and symptoms since March 2020, and measured the patients’ height and weight. SARS-CoV-2 serologic tests were performed on residual patient serum samples. We used the BioSys Plus™ COVID-19 IgM/IgG Rapid Test, a lateral flow immunoassay test device, which has a sensitivity of 93.5% and specificity of 100% for IgG and IgM antibodies to SARS-CoV-2 [22]. The test uses a unique combination of SARS-CoV-2 antigen-coated colloidal gold dye particles to detect and differentiate IgG and IgM antibodies to SARS-CoV-2. This test has not been reviewed by the United States Food and Drug Administration.

The BioSys Plus™ COVID-19 IgM/IgG Rapid Test was used according to the manufacturer’s instructions. Following the collection of the venous whole blood sample in a blood collection tube, the tube of blood was well mixed before one drop of whole blood was transferred to the sample well of the test cassette via a dropper, followed by two to three drops of the buffer. The test was read 10 to 15 minutes afterward. The presence of a control line was used to verify that the test was performed correctly. Results were recorded on a data collection form, which was stored securely and subsequently transferred to a password-protected Excel file. Patients were given the opportunity to receive their SARS-CoV-2 antibody test results. Instructions were provided regarding how to proceed after receiving the test result, whether positive or negative.

Statistical analysis

A descriptive analysis was performed, summarizing each participant’s demographics and clinical history. Analysis of variance (ANOVA) was used to test subject demographic effects on the crude SARS-CoV-2 seroprevalence using JMP (version 11.2.0; SAS Institute, Cary, NC). When ANOVA testing indicated significant differences, post-hoc comparisons were run utilizing the Student’s t-test. The seroprevalence was adjusted for the reported sensitivity (93.5%) and specificity (100.0%) of the test. The adjusted seroprevalence and 95% confidence intervals were calculated using R (version 4.1.0, RStudio, Boston, MA), the epiR package (version 2.0.33, Emory, Atlanta, Georgia), and the epi.prev function. Linear regression was used to model the seroprevalence over time using the least squares method for the generalized linear model (R function glm).

## Results

Of the 9,740 patients seen at the HAH laboratory from December 17, 2020 to July 3, 2021, 538 consented to have antibody testing and answer survey questions, and 529 were included in the final analysis. We excluded nine participants who were aged greater than 89 (n=3), who were aged less than one (n=2), and who had results that were not properly recorded (n=4). Three of the tested patients were repeat testers, with one who had been tested three times. These repeat results were included for a final seroprevalence analysis of 533 samples.

The mean age was 41.9 years (range: 2-88). 60% (315/529) of participants were women. 33% (173/533) of participants reported having at least one of the following symptoms at the time the test was performed: fever, chills, cough, shortness of breath, sore throat, runny nose, fatigue, headache, vomiting, diarrhea, anosmia, ageusia. Comorbidities and risk factors for mortality within the participant pool included: hypertension (21%; 111/529), diabetes (8%; 42/529), overweight (28%; 148/529), obesity (26%; 139/529), and current smoker (3%; 13/529). BMI data were not collected for 89 participants, and rare conditions were not recorded.

In the final participant pool, 142 (26.6%) of 533 samples tested positive for either IgM, IgG, or both antibodies to the SARS-CoV-2 virus. Few participants tested positive for only IgM (2%, n=13) or only IgG (0%, n=2). Adjustment for test sensitivity (93.5%) and specificity (100%) resulted in a greater seroprevalence estimate of 28.7% (95% CI 24.9-32.9).

Subjects were classified by biological sex, age groups, and BMI categories. Participants between one and 17 years old exhibited low adjusted seropositivity relative to all other age groups (5.9%, 95% CI 1.6-19.4, n=36; p<0.5) (Table 1). Adults between 18 and 49 years old exhibited greater adjusted seropositivity compared to those 17 years old or less, but lower adjusted seropositivity when compared to those 50 years old and older (24.3%, 95% CI 19.8-29.3, n=335; p<0.5). Those between 50 and 64 (40.6%, 95% CI 30.4-51.8, n=87) and those over 65 years old (46.1%, 95% CI 33.9-59.0, n=70) exhibited greater seropositivity relative to those less than 50 years old, but the adjusted seropositivity of the two older groups was not significantly different from each other (Table 1). Seropositivity differences were not observed between sexes, among BMI categories, or those who had COVID-like symptoms versus asymptomatic at the time of the test (Table 1).

**Table 1:** Seroprevalence (%) comparisons among demographic groups (n=533) Data are n (%) and % (95% CI). *Estimate adjustments for sensitivity (93·5%) and specificity (100%) of the diagnostic test. **n=444 due to BMI data not collected for 89 participants.

	Positive result	Adjusted seroprevalence*
Age, years		
1–17	36 (5·6%)	5·9% (1·6–19·4)
18–49	335 (22·7%)	24·3% (19·8–29·3)
50–64	87 (37·9%)	40·6% (30·4–51·8)
65+	65 (42·7%)	46·1% (33·9–59·0)
Sex		
Female	315 (26·7%)	28·5% (23·6–34·0)
Male	218 (27·1%)	28·9% (23·1–35·6)
BMI**		
Normal (<25)	157 (20·4%)	21·8% (15·9–29·3)
Overweight (>25 and <30)	148 (28·4%)	30·0 (23·2–38·6)
Obese (>30)	139 (28·1%)	30·0 (22·7–38·5)
Symptomatic at time of test		
Yes	173 (23·7%)	25·3% (19·2–32·7)
No	209 (25.8%)	27·6 (21·8–34·4)

**Figure 1:**
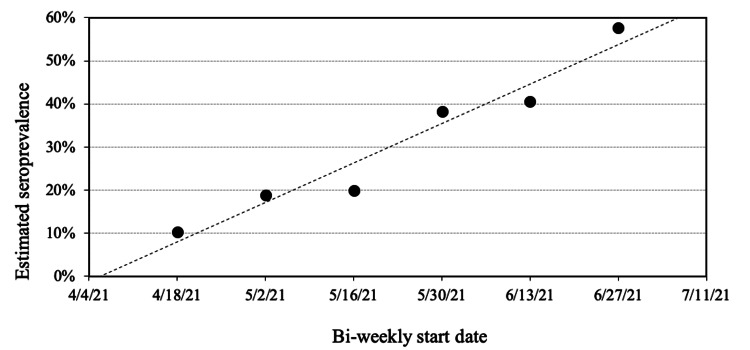
Linear regression of biweekly adjusted seropositivity rate (%).

A linear regression model of the adjusted seroprevalence shows an average bi-weekly increase of 9.2% starting from April 18, 2021 to July 10, 2021, with a strong fit (r2 = 0.9435, p-value = 0.0012) (Figure 1). The first bi-weekly adjusted seroprevalence was 10.3% (95% CI 5.1-19.8) and the last bi-weekly adjusted seroprevalence was 57.6% (95% CI 37.9-76.2). The weeks prior to April 18 were not included in the bi-weekly analysis due to inadequate sample volume.

## Discussion

**Figure 2:**
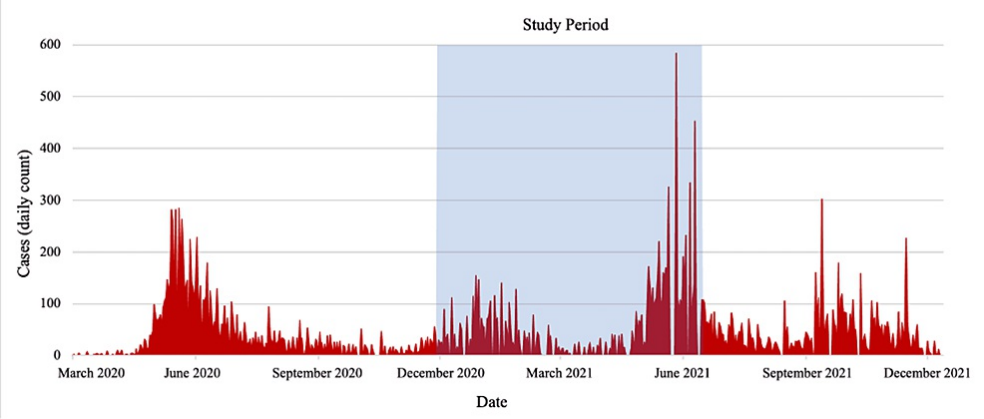
Daily cases in Haiti as reported by the World Health Organization Coronavirus (COVID-19) Dashboard. Blue shaded area represents the period during which study was performed.

The findings from this convenience sampled cohort demonstrate that the seroprevalence of IgG or IgM anti-SARS-CoV-2 antibodies was approximately 28.7% in patients visiting the HAH laboratory in Carrefour, Haiti between the months of December 2020 and July 2021. A vast majority of participants (99.6%; 531/533) were IgM positive, suggesting recent infection. The study was performed during the third upswing in cases seen in Haiti, which is reflected in the linear regression (Figure 1), during which the adjusted seroprevalence rose from 10.3% to 57.6%. The first wave peaked in June 2020 after the introduction of COVID to Haiti in March 2020, followed by a second smaller wave that peaked in January 2021 (Figure 2) [23].

This study describes one of the first estimates of the seroprevalence of anti-SARS-CoV-2 antibodies in Haiti. Tagliamonte et al. published a seroprevalence of 39% (n=4550) after the first wave using data collected by GHESKIO Centers, the only institution other than the Haitian National Laboratory authorized to perform polymerase-chain-reaction testing for COVID in Haiti [21]. Additionally, a subgroup (n=236) of the GHESKIO study group had a PCR-confirmed history of COVID, and of these, only 56% remained seropositive on serologic antibody testing three to six months later. The data from our study are consistent with the findings of Tagliamonte et al. and add to the body of evidence that actual case numbers in Haiti were significantly higher than those that were being reported. The findings from our study suggest that, assuming a Haitian population of 11.4 million people [24], at least 3 million have already been infected with COVID, a number that is grossly larger than the 18,844 confirmed cases reported as of July 3, 2021, the last day of data collection in our study [5]. Given that testing was being rationed for the most critical cases due to extremely inadequate testing resources, it is likely that the officially reported COVID-19 statistics underrepresent actual numbers by several orders of magnitude. During the pandemic, there have been reports of a number of outbreaks of undiagnosed febrile illnesses sweeping across the country, but due to a lack of testing resources and stigmatization against being tested for COVID-19 in Haiti, these outbreaks were not confirmed to be caused by COVID-19. Of note, the data for this study were collected prior to the arrival of vaccines in Haiti on July 14, 2021. Therefore, the antibodies detected were due to prior infection with COVID, not as a result of vaccination.

A variety of theories have been proposed as to why Haiti and some other low-to-middle income countries have not been as severely affected by COVID-19 as expected [25]. One of these factors may be age. The median age in Haiti is 24.0 years, versus 28.0 years in the Dominican Republic and 38.3 years in the United States [26]. Young people are less prone to severe illness or death from COVID-19. Younger populations also tend to have fewer comorbidities when compared with older populations. The lower prevalence of known risk factors for severe or fatal COVID-19 infections such as chronic heart or lung disease may also contribute to the limited impact of COVID-19 in Haiti. Additionally, many of those with severe chronic diseases may die prematurely in low-income settings such as Haiti due to a lack of infrastructure and available resources for treating advanced illnesses. For example, long-term care facilities, often hotspots for infection in higher-income countries due to a concentrated population of vulnerable individuals, are not common in Haiti.

Another theory for the lower rates of severe COVID-19 infections in low-income countries, such as Haiti and several countries in sub-Saharan Africa, is the partial immunity that may be attained through exposure to other coronaviruses and a variety of other pathogens [25]. Both antibody and T-cell-mediated immunity may contribute to an enhanced immune response against COVID-19, leading to lower morbidity and mortality.

Adequate ventilation and open-air environments have been thought to reduce the risk of COVID-19, which may also at least partially explain the decreased impact of the pandemic in rural areas. In Haiti, social distancing measures have been largely disregarded and strict adherence to mask wear has not been followed. Urban marketplaces and public transportation are very crowded, and few alternatives exist. However, these spaces are typically outdoor areas with ample ventilation. The results of our study suggest that activities of daily living in well-ventilated spaces without adherence to infection control measures have not prevented COVID-19 infection but may have influenced mortality and morbidity. As the pandemic evolves, measures are being taken around the world to limit mortality and morbidity rather than put all efforts into preventing the spread of disease. While the outdoor conditions of life in Haiti have not necessarily prevented the spread of disease, they have likely played a role in limiting its severity.

Initial theories suggested that Haiti had a lower incidence of COVID compared to the neighboring Dominican Republic because of the lower numbers of people potentially importing COVID into the country. The Dominican Republic is a popular tourist destination, recording more than 7.5 million international tourist arrivals in 2019, compared with 938,000 in Haiti [27], which had a U.S. Department of State Level 4 travel advisory preceding the pandemic due to civil unrest and crime. This is not necessarily supported by the findings in our study, which show an antibody seroprevalence rate that is much higher than in certain areas of the United States and may indicate rates of exposure equal to or greater than those found in the Dominican Republic [13]. In addition, an initial COVID wave occurred during a strict lockdown of all international travel to Haiti and tapered off as travel restrictions were released in July 2020. The findings of this study highlight the resilience of the Haitian people, who have been infected by the SARS-CoV-2 virus with seemingly minimal symptoms and an exceptionally low death rate. Certainly, lack of access to testing has contributed to this but it does not offer a complete explanation of the dramatic low clinical impact of the disease in Haiti. The death rate from COVID-19 in Haiti is also likely underreported, but again, not completely accounted for based on reporting discrepancies alone.

The severe lockdown enacted by the government of Haiti was well intended but failed to consider how food insecurity, loss of maternal and child lives, and sociopolitical instability were likely to be exacerbated by taking such aggressive actions to control the virus. Understanding transmissibility, clinical response, and death rates from COVID-19 in Haiti can help guide public policy in Haiti and other countries. Our study supports customizing preventative measures for COVID-19 based on differences in immune response and potential economic impact.

The results of this study may emphasize lifestyle factors that portend a more robust immune response. Genetic factors may also be implicated. These influences should be considered as the pandemic response shifts from exposure prevention measures to the minimization of mortality and morbidity. Lack of comorbidities, youth, and vaccination is known to decrease the severity of disease, but other lifestyle and/or genetic factors likely play an important role [25].

Our study had several limitations. Due to the high degree of stigmatization against COVID, random sampling of the community was not practical. Because of this, we chose to utilize a convenience sampling approach, so we are unable to extrapolate our findings to the population of Haiti due to potential selection bias. The participants who were invited to take part in the research study were patients who needed a blood draw ordered by a physician for any number of reasons, including labs ordered during a wellness exam, an urgent visit for acute complaints, or a follow-up visit for chronic diseases. The rates of comorbidities in the sample population were similar to those found in other studies and reports about Haiti [28-29]. But the mean age of the cohort (41.9 years) is significantly higher than the median age in Haiti of 24.0 years [26]. Women were moderately over-represented in this study compared with the population of Haiti as a whole. Recruitment for the study gained momentum over time as the research team fine-tuned their communication about the study to the patients. This increased uptake coincided with the third wave of infections starting to sweep across the country in June 2021, which could have introduced another potential for selection bias.

## Conclusions

In conclusion, our study demonstrates that SARS-CoV-2 seropositivity rates are high in Haiti despite a low number of reported cases and deaths. This study, which took place during the upswing of the third wave of infections in May to June of 2021, found a seroprevalence of 26.6% with a linear regression model demonstrating a rising rate of seroprevalence mirroring the epidemic curve, though convenience sampling limits generalizability. Reasons for the limited clinical impact are unclear but are important for public policy and may elucidate lifestyle factors that may protect against COVID-19. Public health policies enacted in settings like Haiti should not necessarily mimic those of other countries with higher prevalence, especially given the fragile infrastructure and economic challenges of a population which continues to brave an unending barrage of significant challenges.
